# Translation, cross-cultural adaptation, reliability and construct validity of the Dutch Oxford Knee Score – Activity and Participation Questionnaire

**DOI:** 10.1186/s12891-021-04521-0

**Published:** 2021-08-17

**Authors:** Malou E. M. te Molder, Johanna E. Vriezekolk, Menno R. Bénard, Petra J. C. Heesterbeek

**Affiliations:** 1 Department of Research, Sint Maartenskliniek, PO Box 9011, 6500 GM Nijmegen, The Netherlands; 2 Department of Orthopaedic Surgery, Radboud University Medical Center, Nijmegen, The Netherlands

**Keywords:** Oxford knee score – activity and participation questionnaire, Dutch version, Total knee arthroplasty, Patient-reported outcome measure, Translation, Validation

## Abstract

**Background:**

Patients undergoing total knee arthroplasty (TKA) tend to be younger and tend to receive TKA at an earlier stage compared to 20 years ago. The Oxford Knee Score – Activity and Participation (OKS-APQ) questionnaire evaluates higher levels of activity and participation, reflecting activity patterns of younger or more active people. The purpose of this study was to translate the OKS-APQ questionnaire into Dutch, and to evaluate its measurement properties in pre- and postoperative TKA patients.

**Methods:**

The OKS-APQ was translated and adapted according to the forward–backward translation multi step approach and tested for clinimetric quality. Floor and ceiling effects, structural validity, construct validity, internal consistency and test–retest reliability were evaluated using COSMIN quality criteria. The OKS-APQ, the Oxford Knee Score (OKS), the Short Form-36 (SF-36), a Visual Analogue Scale (VAS) for pain and the Forgotten Joint Score (FJS) were assessed in 131 patients (72 preoperative and 59 postoperative TKA patients), and the OKS-APQ was administered twice in 50 patients (12 preoperative and 38 postoperative TKA patients), after an interval of minimal 2 weeks.

**Results:**

Floor effects were observed in preoperative patients. Confirmatory factor analyses (CFA) indicated a good fit of a 1-factor model by the following indices: (Comparative Fit Index (CFI): 0.97, Tucker-Lewis Index (TLI): 0.96 and Standardized Root Mean Square Residual (SRMR): 0.03). Construct validity was supported as > 75% of the hypotheses were confirmed. Internal consistency (Cronbach α’s from 0.81 to 0.95) was good in the pooled and separate pre- and postoperative samples and test–retest reliability (Intraclass Correlation Coefficients (ICCs) from 0.63 – 0.85) were good in postoperative patients and moderate in preoperative patients. The standard Error of Measurements (SEMs) ranged from 8.5 – 12.2 and the Smallest Detectable Changes in individuals (SDC_ind_) ranged from 23.5 – 34.0 (on a scale from 0 to 100).

**Conclusions:**

Preliminary findings suggest that the Dutch version of the OKS-APQ is reliable and valid for a Dutch postoperative TKA patient sample. However, in a preoperative TKA sample, the OKS-APQ seems less suitable, because of floor effects and lower test–retest reliability. The Dutch version of the OKS-APQ can be used alongside the OKS to discriminate among levels of activity and participation in postoperative patients.

## Background

The Oxford knee score (OKS) questionnaire is a validated patient-reported outcome measure (PROM), developed for patients undergoing Total Knee Arthroplasty (TKA) [1]. The OKS was developed in 1998 to reflect patients’ perception of knee pain and functional impairment after TKA [1]. Nowadays, patients undergoing TKA tend to be younger and receive TKA at an earlier stage compared to 20 years ago [2, 3]. According to the latest annual report of the Dutch Arthroplasty Register (LROI), 17% of the primary TKAs were performed in patients younger than 60 years old [4]. Younger patients with an active lifestyle, have higher expectations of the outcome after the procedure [5, 6]. Patients want to stay active and engaged in their social and recreational activities up to and after retiring [2]. Regaining a higher level of participation in social and recreational activities becomes more important for patients after TKA [2]. This implies that besides pain and disability, higher levels of activity and participation have become an important outcome domain. For that reason, the original OKS was extended with an additional one-dimensional scale, the Oxford Knee Score – Activity and Participation Questionnaire (OKS-APQ), to better monitor changes in activity and participation levels after TKA [5].

While TKA procedures are highly successful because it is proven to relieve pain and to improve function, still a significant proportion of approximately 20% of the patients is not satisfied after surgery [7, 8]. The fulfilment of preoperative patient expectations clearly seems to play an important role in patient satisfaction [7]. Especially in younger patients because they expect to perform better on many activities of daily life, work and leisure time after TKA [6]. Therefore, it is important to use questionnaires that reflect patients’ perception of their quality of life, including activities relevant for younger patients.

Following the Dutch Orthopaedic Association (NOV) TKA guideline (2014), patients undergoing TKA in our hospital complete a standard set of questionnaires (e.g. OKS, KOOS-PS, NRS and EQ-5D) for routine outcome monitoring [9]. This set of questionnaires no longer seems sufficient due to concerns about existing ceiling effects of the OKS and EQ-5D in younger patients. Meaning that highest scores on the OKS and EQ-5D would not necessarily reflect treatment satisfaction in the younger patient group [6]. This has been observed by Dawson et al. and in response they developed the OKS-APQ to extend the OKS [5]. Before the OKS-APQ may be used in Dutch clinical practice for outcome monitoring or used for research purposes in young and active patients, the OKS-APQ needs to be translated into Dutch and the measurement properties need to be examined.

The unidimensional, eight-item OKS-APQ evaluates activity and participation levels (e.g. sports, dancing, and participation in activities with friends and family) that fit activity patterns of younger or more active patients. It consists of four highly valued activities and four items concerning performance and awareness (e.g. timing and adjustments of activities) [5]. Besides the original English version of the OKS-APQ and a Chinese version [10], the questionnaire has not been translated and validated in other languages including Dutch. The original OKS-APQ has shown to be a valuable complement to the OKS, particularly where further detail regarding the levels of activity and participation are required [5].

The present study aimed to translate the OKS-APQ into the Dutch language and to assess the unidimensionality of the instrument, the test–retest reliability, internal consistency, construct validity and floor and ceiling effects, in pre- and postoperative TKA patients [11].

## Methods

We performed a translation of the OKS-APQ into Dutch and prospectively evaluated the measurement properties of the Dutch version. Measurement properties were evaluated using COSMIN quality criteria [11].

### Procedure of translation

The OKS-APQ questionnaire was translated from English to Dutch according to the advised forward–backward translation multi step approach for translation as described by Beaton et al. [12, 13]. First, two independent native Dutch translators (DT1 and DT2) translated the OKS-APQ questionnaire to Dutch (forward translation). A definitive version (V12) was based on consensus within a team of translators, health professionals and researchers. Second, two native English translators (ET1 and ET2), blinded to the original English version by Dawson et al., independently re-translated the Dutch version (V12) into English (backwards translation) [5]. Third, the definitive Dutch version of the OKS-APQ was made after a consensus meeting with the team. During the last step, the comprehensibility and interpretability of the definitive version was pilot-tested in a subset of 5 preoperative and 5 postoperative TKA patients. These patients completed the questionnaire at home and were asked to make notes if they thought a question was difficult to understand. Hereafter, a researcher contacted all 10 patients by telephone to discuss the difficulties and to ascertain the meaning that patients attributed to the OKS-APQ items [14]. Recruiting patients for the pilot test was stopped after 10 patients because no issues regarding the OKS-APQ items were reported or emerged. Therefore, no alterations were made to the instruction or questions.

### Patients

As a rule of thumb, at least 100 patients were required and we aimed to include preoperative and postoperative patients. The preoperative study sample was recruited from the waiting list for TKA. Postoperative patients were selected from the outpatient registry. Inclusion criteria for the study participants were: clinically diagnosed with knee OA, age above 18 years, scheduled for TKA within the next 6 weeks or had undergone TKA between 6 and 12 months ago. Patients unable to speak Dutch and understand Dutch written language were excluded. All patients would undergo or underwent TKA at the department of orthopaedics at the Sint Maartenskliniek in Nijmegen. The study was assessed by the local hospital review committee. No ethical approval was sought for, as this study was not subject to the Dutch medical research involving human subjects act. All patients gave their written informed consent prior to study participation.

### Questionnaires

Besides completing the OKS-APQ, patients completed additional condition-specific questionnaires commonly used in pre- and postoperative TKA patients for hypothesis testing purposes between January 2017 and December 2019. All preoperative patients completed the following four questionnaires: the OKS-APQ, the Oxford Knee Score (OKS) [15], the Short Form-36 (SF-36) [16], and a Visual Analogue Scale for pain [17]. Postoperative patients also completed an additional fifth questionnaire, the Forgotten Joint Score (FJS) [18]. All patients were asked to complete the OKS-APQ questionnaire for a second time, after a minimum of two weeks, which was considered appropriate for the test–retest reliability [11].

#### Oxford knee score—activity and participation (OKS-APQ)

The OKS-APQ eight-item questionnaire was developed to measure higher levels of activity and participation and is recommended to be used to complement the OKS as an additional scale [5]. Items are scored on a five-point Likert scale, ranging from 0 “strongly agree” to 4 “strongly disagree”. Total summary score ranges from 0 to 32, and scores are converted to a 0 to 100 measure [5]. A lower total sum score represents lower levels of activity and participation [5].

#### Oxford knee score (OKS)

The OKS 12-item questionnaire has been developed for patients undergoing TKA to evaluate the patients’ perception of pain and functional impairment in the knee [15]. The Dutch questionnaire consists of 12 questions and it is possible to derive separate OKS pain and function subscales [15]. Responses are scored on a 5-point Likert scale, ranging from 0 “significant disability” to 4 “no problem”, in which the final score is an aggregate, sum score for pain and function [19]. The total scores ranges from 0 to 48; a lower OKS sum score represents poor function and more pain. The Dutch OKS has good measurement properties [15], however ceiling effects were demonstrated in postoperative patients [20, 21].

#### MOS short form 36 (SF-36v2)

The Dutch SF-36 version 2 is a 36-item questionnaire assessing health-related Quality of Life (QoL). It consists of eight dimensions that are aggregated to two summary scores: Physical Component Score (PCS) and Mental Component Score (MCS) (both 0–100) [16]. The SF-36 is widely used and has shown to be reliable and valid in the Dutch general population [16, 22, 23]. A lower score represents a lower level of QoL [15].

#### Visual analogue scale for pain (VAS pain)

The Dutch VAS for pain is a single item scale assessing the intensity of pain in the knee during the past 2 to 3 days. The 100-mm VAS is simple to use, and has already been applied in different populations and settings [17]. The score varies from 0 (no pain), to 100 (worst pain). It has shown to be valid and reliable [17].

#### Forgotten joint score (FJS-12)

The Dutch 12-item Forgotten Joint Score (FJS-12) questionnaire evaluates the patients’ ‘joint awareness’ during activities of daily living (i.e. stair climbing, walking and gardening). The responses were scored on a five-point Likert scale, ranging from 0 “never” to 4 “mostly”. Item scores were summed and converted to a 0 to 100 scale, a low total sum score reflects that the patient is not able to forget the affected/replaced joint during activities of daily living [18]. The Dutch FJS-12 has shown to be a reliable and valid questionnaire [24].

### Methodological testing & statistical analysis

Kolmogorov Smirnov test was used to test the normality of the OKS-APQ items, OKS-APQ total score and other PROM total scores. Descriptive statistics were used to summarize the data; mean and standard deviation (SD) or median (25^th^ – 75^th^ percentile) for continuous variables and counts and percentages for categorical variables, and to investigate the frequencies of missing data. All statistical analyses were performed in STATA version 13.0 (StataCorp, College Station, Texas). A *P*-value < 0.05 was considered statistically significant for all analyses.

#### Validity

Validity is the degree to which the Dutch OKS-APQ measures the construct(s) it purports to measure. To evaluate validity, floor and ceiling effects, structural validity and construct validity were measured.

#### Floor & ceiling effects

Another quality criterion is the absence of floor and ceiling effects. Presence of floor and ceiling effects on the OKS-APQ may influence the test–retest reliability, and construct validity of the questionnaire [25]. Patients with the lowest or highest possible score cannot be distinguished from each other, thus reliability is reduced [25]. Floor and ceiling effects, in pre- and postoperative samples separately, were determined by calculating the number of individuals that obtained the lowest (0) or highest (100) scores possible and were considered present if more than 15% of the patients achieved the highest or lowest total summary score [25]. In addition, floor and ceiling effects on item-level were determined to provide information about the item distribution.

#### Structural validity

Confirmatory factor analyses (CFA) was used to validate the 1-factor structure of the original English version of the OKS-APQ [5]. We examined the comparative fit index (CFI; values ranging from 0.90 to 0.95 indicate an adequate fit and values greater than or equal to 0.95 indicate a good fit), the Tucker-Lewis Index (TLI; values ranging from 0.90 to 0.95 indicate an adequate fit and values greater than or equal to 0.95 indicate a good fit), the root mean square error of approximation (RMSEA; values ranging from 0.05 to 0.08 represent adequate fit and values less than 0.05 indicate good fit) and the standardized root mean squared residual (SRMR; values less than or equal to 0.08 indicate good fit) to assess goodness of fit of this model. CFA was assessed using the pooled sample (pre- and postoperative patients).

#### Construct validity

Validity is the degree to which the OKS-APQ measures the construct it supposes to measure. Since there is no gold standard in the measurement of PROMs, validity was measured as construct validity [25]. Construct validity refers to the extent to which the OKS-APQ was related to other measures based on theoretically derived, predefined hypotheses. Construct validity was supported when at least 75% of the results are in accordance with the predefined hypotheses (Table 1) [25]. Construct validity was expressed by assessing Pearson correlation coefficients or the nonparametric Spearman’s correlation coefficients [25]. The strength of the correlations was interpreted as “weak” (*r* = 0.10 – 0.30), “moderate” (*r* = 0.31 – 0.50) or “strong” (*r* = 0.51 – 1.00) [26]. Predefined hypotheses were formulated for the pooled and separate pre- and postoperative samples.

**Table 1:** Predefined hypotheses for evaluating the construct validity of the Dutch OKS-APQ

**Hypothesis**
A. Strong positive correlation (*r* > 0.50):
1. A strong positive correlation between OKS-APQ and OKS (pooled, pre- and postoperative patients);
2. A strong positive correlation between OKS-APQ and FJS (in postoperative patients);
3. A strong positive correlation between OKS-APQ and PCS (SF-36) (pooled, pre- and postoperative patients);
B. Moderate to strong negative correlation (*r* > 0.31):
4. A moderate to strong negative correlation between OKS-APQ and VAS pain (pooled, pre- and postoperative patients);
C. Weak to moderate positive correlation (*r* 0.10 – 0.50):
5. A weak to moderate positive correlation between OKS-APQ and MCS (SF-36) (pooled, pre- and postoperative patients);

*Abbreviations*: *OKS-APQ* Oxford knee score – Activity & Participation Questionnaire, *FJS* Forgotten joint score, *OKS* Oxford knee score, *SF-36* 36-Item Short Form Health Survey Questionnaire, *VAS for pain* Visual Analogue Scale, *PCS* Physical Component Score, *MCS* Mental Component Score

#### Reliability

Reliability is the degree to which the Dutch OKS-APQ is free from measurement error. To evaluate reliability, internal consistency, test–retest reliability, the measurement error and the smallest detectable change were calculated.

#### Internal consistency

Internal consistency is a measure to evaluate to what extent the eight items of the Dutch OKS-APQ refer to the same underlying construct [25]. Internal consistency of the Dutch version of the OKS-APQ was determined by calculating the Cronbach’s alpha [25]. A Cronbach’s alpha between 0.7 and 0.9 for the eight items of the OKS-APQ indicates good internal consistency [25]. The Cronbach’s alpha was measured on the pooled sample and the separate pre- and postoperative samples.

#### Test–retest reliability

Test–retest reliability involves the degree to which the results of the Dutch OKS-APQ are consistent across repeated measurements [25]. To evaluate the reliability of the Dutch OKS-APQ, we calculated intraclass correlation coefficients (ICCs) with a 95% confidence interval (95% CI). In addition, we provided the different variance components to show the systematic differences between the two timepoints in preoperative and postoperative patients. More specific, we used the ICC two-way random effects model type agreement to measure the reliability [25]. An ICC equal to and larger than 0.7 is generally accepted as good [25]. ICCs were calculated for the separate pre- and postoperative samples.

#### Measurement error & smallest detectable change

The measurement error is the systematic and random error of a participant’s score that is not attributed to true changes in the construct to be measured [11]. The standard error of measurement (SEM) was calculated using the square root of the error variance [14, 25].

The smallest detectable change (SDC) reflects the smallest individual change in score that can be interpreted as a real change in one individual (SDC_ind_). This was calculated by the SEM * 1.96 * √2 [14, 25]. The SDC_ind_ can be divided by √n (*n* = sample size) to calculate the SDC in a group of patients (SDC_group_) [14, 25]. SEM and SDC were calculated for the separate pre- and postoperative samples.

## Results

### Demographic data

A total of 131 patients were included, with mean age 66.3 (9.4) years, of whom 72 were preoperative patients with OA prior to TKA, and 59 were postoperative patients ≥ 6 months after TKA (Table 2). Both the pooled data and the separated pre- and postoperative samples were not normally distributed (*p* < 0.05). The missing values per item and for the total scores ranged from: 0 to 5.34% missing values, with the latter only for VAS pain. All missing items on the OKS-APQ (Table 3), OKS and SF-36 were imputed as recommended with patient-specific mean values of completed items. Because 10% missing data for a variable is considered acceptable [27], we performed the analyses without further evaluation or adjustment of the other variables.

**Table 2:** Patient characteristics

***Sociodemographic***	**Pooled sample** **(*n* = 131)**	**Preoperative sample (*n* = 72)**	**Postoperative sample** **(*n* = 59)**
Age; mean (SD),(yr)	66.3 (9.4)	66.2 (9.3)	66.4 (9.6)
***Self-report measures; median (25***^***th***^*** – 75***^***th***^*** percentile)***			
OKS—Activity & Participation (OKS-APQ) *(scale 0–100)*	21.9 (6.3–56.3)	10.9 (0–23.4)	62.5 (25–84.4)
Oxford Knee Score (OKS) *(scale 0–48)*	29 (20–39)	22 (15–29)	39 (30–44)
VAS Pain *(scale 0–100)*	30 (10.5–63.5)	59 (31–74)	11 (4–28)
*Quality of life*			
Physical component (SF-36-PCS) *(scale 0–100)*	34.1 (27.8–40.8)	30.6 (25.8–34.8)	39.8 (33.6–46.8)
Mental component (SF-36-MCS) *(scale 0–100)*	52.8 (42.5–57.2)	50.6 (41.7–56.4)	53.7 (48.0–57.3)
Forgotten Joint Score (FJS) *(scale 0–100)*	*NA*	*NA*	37.5 (14.6–60.4)

*SD* indicates standard deviation, *OKS APQ* Oxford Knee Score—Activity and Participation Questionnaire, *OKS* Oxford Knee Score, *VAS* Visual Analogue Scale, *SF-36* 36-item Short Form Health Survey Questionnaire, *SF-36-MCS* Mental Component Score, *SF-36-PCS* Physical Component Score, *FJS* Forgotten Joint Score

**Table 3:** Characteristics of the Dutch OKS-APQ

**Items**	**Item-Total Correlation (pooled sample)**	**Missing, n (%) (pooled sample)**	**Floor, n (%) (preoperative sample)**	**Ceiling, n (%) (preoperative sample)**	**Floor, n (%) (postoperative sample)**	**Ceiling, n (%) (postoperative sample)**
It is a problem for me to do activities (e.g. sports, dancing, walking) to the level I want, because of my knee	0.84	0 (0.0%)	64 (89%)	1 (1.4%)	19 (32.2%)	7 (11.9%)
It is a problem for me to carry heavy things (e.g. items at work, shopping or a child), because of my knee	0.83	0 (0.0%)	41 (56.9%)	1 (1.4%)	12 (20.3%)	15 (25.4%)
I need to modify my work or everyday activities, because of my knee	0.92	4 (3.1%)	42 (60%)	3 (4.3%)	11 (19.3%)	18 (31.6%)
I need to plan carefully before going out for the day because of my knee (e.g. taking painkillers, using a knee brace or checking that there will be places to sit down)	0.89	0 (0.0%)	44 (61.1%)	2 (2.8%)	9 (15.3%)	27 (45.8%)
It is a problem for me to fully take part in activities with friends and family, because of my knee	0.85	0 (0.0%)	34 (47.2%)	1 (1.4%)	11 (18.6%)	18 (30.5%)
It is a problem for me to walk at the pace I would like, because of my knee	0.88	0 (0.0%)	62 (86.1%)	10 (13.9%)	16 (27.1%)	12 (20.3%)
It is a problem for me to twist or turn, as my knee may give way or be painful	0.87	0 (0.0%)	46 (63.9%)	3 (4.2%)	10 (17%)	23 (39%)
It is a problem for me that I need to take longer to do everyday activities, because of my knee	0.88	1 (0.8%)	29 (40.9%)	8 (11.3%)	7 (12.1%)	18 (31%)

### Floor & ceiling effects

Floor effects were observed for the individual items and summary score of the OKS-APQ in the preoperative patient sample (Table 3). Twenty one patients (29.2%) scored the lowest level of activity and participation. No ceiling effect was observed for the summary score and individual items. In the postoperative patient sample, no floor and ceiling effects were observed for the summary score. On item level, both floor and ceiling effects were observed, however responses were much more evenly distributed (Table 3).

### Structural validity

CFA indicated a good fit of a 1-factor model by the following indices: CFI: 0.97, TLI: 0.96, and SRMR: 0.03. However, RMSEA: 0.11, was greater than 0.08.

### Construct validity

Construct validity was assessed with Spearman’s rho correlations and showed a strong positive correlation for the OKS in both pre- and postoperative patients and a strong positive correlation for the FJS-12 and PCS of the SF-36 in postoperative patients (Table 4). The OKS-APQ showed a moderate to strong negative correlation for the VAS pain and a weak to moderate positive correlation for the MCS of the SF-36 in both pre- and postoperative patients.

**Table 4:** Construct validity of the Dutch OKS-APQ

**Predefined Hypothesis**	Spearman correlation* (*r*)		
	**Pooled sample**	**Preoperative sample**	**Postoperative sample**
1. A strong positive correlation between OKS-APQ and OKS;	0.83	0.63	0.80
2. A strong positive correlation between OKS-APQ and FJS;	NA	NA	0.74
3. A strong positive correlation between OKS-APQ and PCS (SF-36);	0.65	0.40	0.59
4. A moderate to strong negative correlation between OKS-APQ and VAS pain;	*–*0.68	–0.43	–0.63
5. A weak to moderate positive correlation between OKS-APQ and MCS (SF-36);	0.47	0.50	0.40

*Abbreviations*: *OKS-APQ* Oxford knee score – Activity & Participation Questionnaire, *OKS* Oxford knee score, *FJS* Forgotten joint score, *SF-36* 36-Item Short Form Health Survey Questionnaire, *VAS for pain* Visual Analogue Scale, *PCS* Physical Component Score, *MCS* Mental Component Score

^*^All correlations *P* < 0.001

### Internal consistency

The item-total correlations were calculated for each item (Table 3). Internal consistency was appropriate; Cronbach alpha values exceeded 0.70 for the pooled and separate samples of pre- and postoperative patients (Table 5).

**Table 5:** Reliability of the Dutch OKS-APQ

**Study sample**	**Pooled sample (*n* = 131)**	**Preoperative sample (*n* = 72)**	**Postoperative sample (*n* = 59)**
Cronbach α	0.95	0.81	0.95
**Test–retest sample**		**Preoperative sample (*n* = 12)**	**Postoperative sample (*n* = 38)**
OKS-APQ test *median (25*^*th*^*–75*^*th*^* percentile)*		9.38 (0.00–29.69)	67.19 (27.34–82.03)
OKS-APQ retest *median (25*^*th*^*–75*^*th*^* percentile)*		15.63 (0.78–28.13)	62.50 (21.88–78.13)
ICC (95% CI)		0.63 (0.10–0.88)	0.85 (0.72–0.92)
Variance components			
σ^2^_p_		125.62	820.31
σ^2^_o_		0.00	2.08
σ^2^_residual_		72.02	147.49
SEM		8.49	12.23
SDC_ind_		23.53	34.00
SDC_group_		6.79	5.52

σ^2^_p_: The variance of the patients (i.e. the systematic differences between the ‘true’ scores of the patients; σ^2^_o_: Variance due to systematic differences between observers/timepoints; σ^2^_residual_: Residual variance (i.e. random error variance)

*ICC* Intraclass correlation coefficient, *SEM* Standard error of measurement, *SDC*_*ind*_ Smallest detectable change in one individual, *SDC*_*group*_ Smallest detectable change in a group

### Test–retest reliability

Fifty patients (12 preoperative and 38 postoperative patients) completed the questionnaires for a second time, after a minimum of two weeks. The median scores (25^th^ – 75^th^ percentile) for the test and retest of the OKS-APQ, the ICCs and variance components are presented in Table 5. The OKS-APQ showed good test–retest reliability in the postoperative samples with an ICC of 0.85. The ICC in the preoperative sample was lower with smaller between-subject variability in preoperative patients (Table 5).

### Measurement error & smallest detectable change

SEM, SDC_ind_ and SDC_group_ in the pre- and postoperative patients are presented in Table 5.

## Discussion

In general, the Dutch OKS-APQ indicated to be an understandable, reliable and valid unidimensional PROM to assess activity and participation levels in post-operative TKA patients. No floor and ceiling effects were observed for the summary score of the OKS-APQ in postoperative patients. However, floor effects were observed in preoperative patients indicating that the Dutch OKS-APQ is not able to discriminate among the lowest levels of activity and participation in the preoperative situation solely based on the OKS-APQ. Furthermore, internal consistency was good in the pooled and separate samples. Test–retest reliability was good in the postoperative sample, however, was moderate in the preoperative sample. In the overall sample, structural validity indicated satisfactory 1-factor model fit.

### Cross-cultural translation

The cross-cultural translation and adaptation procedure in this study yielded a clear, understandable Dutch version of the OKS-APQ. Content validity, including the relevance and comprehensiveness was not evaluated in this study. Likewise, content validity ratio (CVR) and content validity index (CVI) were not determined. Witjes et al., however, showed that the OKS-APQ was rated as an important and relevant questionnaire for younger Dutch TKA patients [6]. Since content validity is an important measurement property according to the recent COSMIN study design checklist for patient-reported outcome measurement instruments [28], further investigation of the Dutch OKS-APQ is advised to evaluate its content validity with patients and experts.

### Floor & ceiling effects

In general, the patterns of observed floor and ceiling effects of the Dutch OKS-APQ for the summary score and at item level were consistent with the original OKS-APQ and the Chinese version of the OKS-APQ [5, 10]. The floor effects found in the preoperative sample might be explained by the fact that these patients were awaiting a TKA and therefore report severe complaints/functional limitations. In the postoperative sample both floor and ceiling effects were present at item level, that can be explained by the varying rehabilitation course after TKA. Some of these patients were still rehabilitating after 6 months, while others were already fully recovered.

### Reliability and structural validity

Confirmatory factor analysis of the Dutch OKS-APQ in our pooled sample of pre- and postoperative patients confirmed the unidimensional structure of the original OKS-APQ [5] as was reflected by good fit indices. Nevertheless, as we can not rule out bias by pooling the data of pre- and postoperative patients, it is important to replicate these findings in larger, separate pre- and postoperative samples. Furthermore, in line with the original and Chinese version of the OKS-APQ, the internal consistency of the Dutch OKS-APQ was good for the pooled and separate sample of pre- and postoperative patients. The test–retest reliability was good for the postoperative sample (ICC = 0.85). In contrast to the Chinese OKS-APQ validation findings that showed an excellent test–retest reliability (ICC = 0.94) in a sample of 30 preoperative patients, we found a moderate test–retest reliability in the preoperative sample (ICC = 0.63). The ICC in the preoperative patient sample was lower than the ICC of the postoperative patient sample which may be explained by the small preoperative sample size in our study, and in turn, the smaller between-subject variability in preoperative patients (see Table 5). Since the ICC is a relative measure depending on both the between-subject variability and test–retest variability similar test–retest variability in combination with smaller between-subject variability resulted in a lower ICC value. Since a sample size of at least 50 patients is recommended for examination of the test–retest reliability of a health measurement instrument [25], further investigation of the Dutch OKS-APQ in larger pre- and postoperative samples is recommended to firmly establish its test–retest reliability.

### Construct validity

The construct validity of the Dutch OKS-APQ was confirmed as more than 75% of our hypotheses were supported. In line with other research [5, 10], the Dutch OKS-APQ strongly correlated with knee specific questionnaires (e.g. OKS and AKSS), and the general SF-36 physical component score. Overall, this suggests that the OKS-APQ, OKS, AKSS and the physical component score of the SF-36 measure similar constructs.

### Clinical implications

For clinical practice, this study shows that the Dutch OKS-APQ is able to discriminate among postoperative patients whereas ceiling effects were previously found for the OKS in postoperative patients [20, 21]. The developers of the OKS-APQ recommend to use the OKS-APQ to complement the OKS as an additional scale [5]. Caution in interpretation of preoperative OKS-APQ evaluation is warranted because of floor effects found in preoperative patients. Evidently, preoperative scores are needed to evaluate effects of surgical interventions as TKA. In addition, the OKS-APQ may provide support for transferring patients to transmural care (e.g. physiotherapy or social work) when patients are still not satisfied with the prosthesis because of problems in social participation and recreational activities including sports. This may be subject for future investigations.

### Limitations

Limited by our cross-sectional study design and small group sample sizes, several measurement properties of the Dutch OKS-APQ could not be evaluated. Further validation studies in larger samples are recommended to more extensively evaluate the content validity (e.g. exploring the relevance and comprehensiveness with patients and experts), structural validity of the OKS-APQ (e.g. testing structure equivalence of the Dutch OKS-APQ in pre- and postoperative TKA patients separately), the reliability and precision of the OKS-APQ (e.g. test–retest reliability in larger pre- and postoperative samples and differential item functioning using item response modelling), responsiveness (e.g. testing the validity of change scores of the Dutch OKS-APQ), interpretability (e.g. by relating the SDC and SEM to the minimal important change (MIC)) and predictive validity. Furthermore, our findings were based on a sample of patients who were treated in a specialized hospital, this should be taken into account when generalizing to other samples or settings.

## Conclusion

Preliminary findings suggest that the Dutch version of the OKS-APQ is reliable and valid for a Dutch postoperative TKA patient sample. However, in a preoperative TKA sample, the OKS-APQ seems less suitable, because of floor effects and lower test–retest reliability. The Dutch version of the OKS-APQ can be used alongside the OKS to discriminate among levels of activity and participation in postoperative patients.

## Data Availability

The datasets used during the current study are available from the corresponding author on reasonable request.

## Abbreviations

OA: Osteoarthritis
TKA: Total knee arthroplasty
PROMs: Patient-reported outcome measures
QoL: Quality of life
VAS: Visual analogue scale
OKS: Oxford knee score
OKS-APQ: Oxford Knee Score – Activity & Participation Questionnaire
FJS: Forgotten Joint Score
SF-36: 36-Item Short Form Health Survey Questionnaire
MCS: Mental Component Score
PCS: Physical Component Score
AKSS: American Knee Society Score
EQ-5D: EuroQol 5D
CFA: Confirmatory factor analyses
ICC: Intraclass correlation coefficient
SEM: Standard error of measurement
SDC: Smallest detectable change
MIC: Minimal important change
CVI: Content validity index
CVR: Content validity ratio
